# Internal blood loss in fatal liver lacerations – determining lethality from relative blood loss

**DOI:** 10.1007/s00414-024-03323-y

**Published:** 2024-09-04

**Authors:** Sandra Holmgren, Torfinn Beer

**Affiliations:** Department of Community Medicine and Rehabilitation/Forensic Medicine, Umeå University, P.O. Box 7616, Umeå, SE-907 12 Sweden

**Keywords:** Forensic, ATLS^®^, Blood loss, Hypovolemic shock, Measurement error, Reference values

## Abstract

Certificates of medical evidence are often used to aid the court in assessing the cause and severity of a victim’s injuries. In cases with significant blood loss, the question whether the bleeding itself was life-threatening sometimes arises. To answer this, the volume classification of hypovolemic shock described in ATLS^®^ is commonly used as an aid, where a relative blood loss > 30% is considered life-threatening. In a recent study of deaths due to internal haemorrhage, many cases had a relative blood loss < 30%. However, many included cases had injuries which could presumably cause deaths via other mechanisms, making the interpretation uncertain. To resolve remaining ambiguity, we studied whether deaths due to isolated liver lacerations had a relative blood loss < 30%, a cause of death where the mechanism of death is presumably exsanguination only. Using the National Board of Forensic Medicine autopsy database, we identified all adult decedents, who had undergone a medico-legal autopsy 2001–2021 (*n* = 105 952), where liver laceration was registered as the underlying cause of death (*n* = 102). Cases where death resulted from a combination of also other injuries (*n* = 79), and cases that had received hospital care, were excluded (*n* = 4), leaving 19 cases. The proportion of internal haemorrhage to calculated total blood volume in these fatal pure exsanguinations ranged from 12 to 52%, with 63% of cases having a proportion < 30%. Our results lend further support to the claim that the volume classification of hypovolemic shock described in ATLS^®^ is inappropriate for assessing the degree of life-threatening haemorrhage in medico-legal cases.

## Introduction

Certificates of medical evidence regarding a living person’s injuries are often issued by a forensic physician [1] in countries with integrated medicolegal services [2–5]. These certificates include detailed descriptions of injuries and other findings, hypotheses of potential causes as well as a general opinion of the severity of the injuries observed [1, 2].

In sharp force violence, damage to blood vessels can lead to significant blood loss and as such, there are often questions about what amount of blood loss an individual can tolerate and whether a specific amount was by itself life-threatening [2]. One way to assess the life-threatening amount is to assess the relative blood loss (RBL) by calculating the proportion of exsanguinated blood volume to the calculated total blood volume [6]. The volume classifications of hypovolemic shock described in Advanced Trauma Life Support (ATLS^®^) are generally used to support such an argument [7]. ATLS^®^ divides hypovolemic shock into four categories based on vital signs and estimated RBL [7–9]: Haemorrhages of class I-II, with a RBL of 30% or less are considered uncomplicated, while haemorrhages of class III-IV, with losses of 30% or more are considered life-threatening.

To calculate the RBL, it is often assumed that the total blood volume (TBV) is equal to 7% of the body weight, as the average adult blood volume is around 70 ml/kg [10, 11]. This ”7% rule” is not optimal, however, as it does not consider sex, height, or body constitution, factors which have been shown to impact the total blood volume [12]. Examples of formulas which consider BMI/constitution are the Nadler-Hidalgo-Bloch equation [13] and the Lemmens-Bernstein-Brodsky Equation [12].

A recent study has given reason to suspect a simple proportion threshold of lethality might be a method to blunt to be used for medico-legal purposes [6]. The study investigated deaths due to internal haemorrhage and found that a considerable proportion of cases had an RBL of < 30%. Depending on the method used to calculate total blood volume, a RBL of < 30% was found in 28–66% of the fatalities [6]. However, this study included cases with also other potential mechanisms for death other than blood loss only, for example aortic rupture or trauma to the heart and/or lungs, leading to premature decompensation [6]. Hence, the interpretation regarding life-threatening RBLs is somewhat muddied.

We believe that the remaining uncertainty left by the previous study could be somewhat alleviated by studying isolated liver lacerations as these cases likely would not suffer such premature decompensation and as such represent “pure” (and slow) fatal internal haemorrhage. Naturally, such cases are exceedingly rare as most trauma causing liver laceration will also cause other significant injuries. However, the value of assessing the hypothesis that the volume of internal haemorrhage in such presumably slow haemorrhage deaths *could* be below 30% in our opinion outweighs the issue of low sample size.

Our goal in this study is therefore not a strict replication of the index study but to examine whether the general finding that there are internal haemorrhage deaths with low RBL volumes holds also in the purest cases possible. To this end, to further investigate whether deaths caused by internal haemorrhage have RBL lower than what is now considered life-threatening, i.e. <30%, we analyzed deaths where the cause of death was haemorrhage from isolated liver laceration *only*.

## Materials and methods

### Study population

**Fig. 1:**
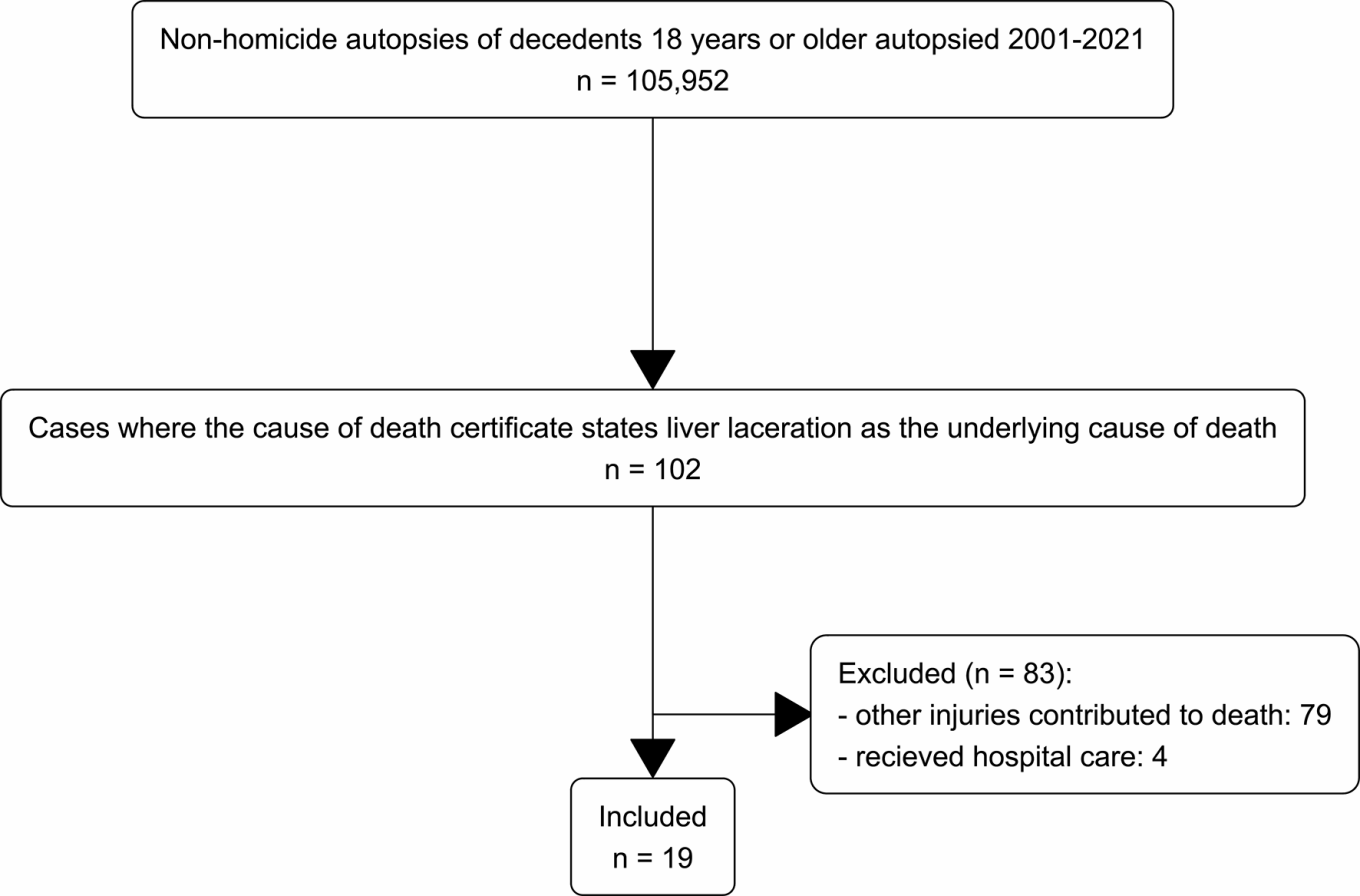
A flow chart showing how cases were selected and excluded/included

In Sweden, medical-legal autopsies are conducted at one of six forensic medicine units of the National Board of Forensic Medicine (NBFM). We searched the NBFM autopsy database from 2001 to 2021 for non-homicide decedents aged 18 years or older who had undergone an autopsy (*n* = 105 952), where the registered underlying cause of death was liver laceration (*n* = 102) (Fig. 1).

We reviewed each autopsy report and excluded cases where the underlying cause of death as recorded in the autopsy report was not liver laceration *only*, e.g. where the cause of death was a combination of injuries (*n* = 79), as well as cases that had received hospital care (*n* = 4), leaving 19 cases (Fig. 1). For these cases, we extracted injuries, height, weight, sex, and measured blood volumes in the body cavities, as well as signs of hypovolemic shock, that is, pale lividity, as well as kidney pallor, lung pallor and/or subendocardial haemorrhage. Further, we also extracted information about known disease and autopsy findings of incidental disease.

### Methods

As in the inciting study we calculated total blood volume using Nadler-Hidalgo-Bloch and Lemmens-Bernstein-Brodsky and Friesen Eqs. [11–13], the latter using Friesen’s altered versions of Janmahasatian’s lean-scale-factor [14] (Table 1). As in the inciting study, we also use the experience-based methods of assuming a total blood volume of 7–8% of body weight [6] (Table 1).

**Table 1:** Formulas used to calculate total blood volume

	Equation
Nadler, Hidalgo, Bloch [12]	♂: *TBV (l)* = (0.3669 × *height* 3 (m) ) + (0.03219 × *weight (kg)*) + 0.6041 ♀: *TBV (l)* = (0.3561 × *height* 3 (m) ) + (0.03308 × *weight (kg)*) + 0.1833
Lemmens, Bernstein, Brodsky [11]	TBV (ml) = weight (kg)$\times\:\frac{70}{\sqrt{\frac{BMI}{22}}}$
Friesen [13] with using his adjusted versions of Janmahasatian’s [14] lean-scaled-factors (LSF)	♂: *TBV (ml) =*$70\times\:(\left(\frac{11432}{6680+216\times\:BMI}\right)\times\:weight\left(kg\right))$ ♀: *TBV (ml) =*$60\times\:(\left(\frac{14148}{8780+244\times\:BMI}\right)\times\:weight\left(kg\right))$
7% rule	*TBV (l) = weight (kg)* × 0.07
8% rule	*TBV (l) = weight (kg)* × 0.08

As noted in the inciting study [6] there are many issues with finding accurate measures of intra-abdominal blood volume (IABV): (I) the presence of both internal and external haemorrhage in many cases, making it difficult, if not impossible, to correctly estimate total blood losses, (II) the impact of resuscitation attempts including causing additional injuries as well the addition of blood or other fluids during resuscitation, which can artificially inflate haemorrhage volume, (III) coexisting pathology limiting haemorrhage volume (as compared to some hypothetical healthy case), (IV) some blood volume being unmeasurable as it is within surrounding tissue, and (V) error in measuring the measurable proportion of internal haemorrhage.

Issue I is alleviated through case selection while issue II can be solved via conducting separate analyses. Regarding the issue of coexisting pathology raised in III we are of the opinion that it is not an error at all but merely a representation of the inherent variation among decedents. However, Issues IV and V are more subtle. It is impossible to estimate the proportion of blood in tissue or to know in each case how much error is associated with the measurement itself. However, in practice, any clinical assessment of blood loss is likely associated with more error than that found in autopsy material. We believe that this, taken together with the fact that we have no reason to believe that error is non-random, means that the error can be safely ignored for the given research question at hand.

However, for the Nadler-Hidalgo-Bloch equation there is a known standard deviation of ± 0.392 l for men and ± 0.413 l for women. As such we chose to implement a Bayesian measurement error model to account for this. In the implementation of a classical Bayesian measurement error model the observed data is treated as samples from a latent distribution of true values [15, 16]. Let TBV^obs^ represent calculated values of total blood volume and let TBV* represent the non-observed true total blood volume without the measurement error $\:\epsilon$ (Eq. 1).

1 $$\:TB{V}_{i}^{obs}\sim normal(TB{V}_{i}^{*},\:\epsilon)$$

Each TBV^*^ value is modelled hierarchically as coming from a common parent distribution with its own hyperpriors $\:\mu\:$ and $\:\sigma\:$ for its mean and SD (Eq. 2, note that the priors here are defined in terms of z-score).

2 $$\begin{aligned} & TB{V}_{i}^{*}\sim\:{\mathrm{normal}}\left(\mu\:,\sigma\:\right)\\& \mu \sim\:{\mathrm{half-normal}}(0,\:5)\\& \sigma \sim {\mathrm{exponential}}\left(1\right)\\\end{aligned}$$

For each case we calculated the RBL by dividing the calculated total blood volume using each method by the observed intra-abdominal blood volume (IABV). For the result of Nadler-Hidalgo-Bloch equation we calculated RBL using both the observed unadjusted values as well as the latent error adjusted values.

For each of the above RBL ratio calculations we used a Bernoulli model to calculate the probability that RBL would be below 30%. For all such calculations we used weak Beta(2,2) priors (Eq. 3).

3 $$\begin{aligned}&{X}=\left\{\begin{array}{ll}1,& if\ RBL<30\%\\ 0,& otherwise \end{array}\right. \\&{X} \sim {\mathrm{Bernoulli}}(\theta)\\& \theta \sim Beta (2,2) \end{aligned}$$

All modeling was done using Stan 2.26 [17] interfaced through Rstan 2.23 [18] as well as with R 4.0.2 [19]. Model results are presented using the mean values from posterior distributions, as well as the 95% highest posterior density intervals (HPDI). All Stan models were run for the standard of 2000 iterations with 1000 discarded as warmup. They were run with 4 chains resulting in 4000 posterior samples for each parameter. The models were run without error and chains converged nicely with $\:\widehat{R}$ values in the range 0.999–1.001.

## Results

### Population

The cases consisted of 14 men and 5 women. The median age was 47 years (range 18–78 years) (Table 2). External causes of injury were traffic crashes (*n* = 9), falls (*n* = 9), and an agricultural accident (*n* = 1). Most cases were either found dead or expired shortly after being found. However, the circumstances in case one suggests that the decedent was injured at home and survived for a week without seeking medical attention. Notably, 6/19 (32%) cases had some form of liver disease (Table 2).

**Table 2:** Cases with isolated liver laceration as the only cause of death and cases with primary cause of death registered as liver laceration, but with additional contributing factors. Intra-abdominal blood volume (IABV) is the measured blood volume in the intra-abdominal cavity during autopsy. Total blood volume (TBV) is calculated using as a product of height, weight, and sex, using the Nadler-Hidalgo-Bloch, Lemmens-Bernstein-Brodsky and Friesen equations as well as a flat 7% and 8% of body weight. Relative blood loss (RBL) is the proportion of IABV to TBV. Signs of hypovolemia (SoH) are any of the following findings noted in the autopsy protocols: pale lividity, as well as kidney pallor, lung pallor and/or subendocardial haemorrhage

Case	Sex	Age	Additional factors	Known or incidental disease findings	SoH	Resuscitation attempt	IABV (litre)	Nadler		Lemmens-Bernstein-Brodsky		Friesen		8% rule		7% rule	
								TBV (litre)	RBL	TBV	RBL	TBV	RBL	TBV	RBL	TBV	RBL
1†	♀	60	-	Steatosis	Kidney pallor	No	1	4.84	21%	5.13	19%	4.52	22%	7.92	13%	6.93	14%
2	♂	35	Fracture of the right humerus. Rib fractures with 150- and 200-ml blood in the right and left pleural cavities, respectively.	-	None	Yes	1.9	5.40	35%	5.39	35%	5.46	35%	6.96	27%	6.09	31%
3	♀	78	Rib fractures.	Atrial fibrillation treated with anticoagulants. Moderate coronary sclerosis	Lung pallor	Yes	2	3.87	52%	4.30	47%	3.71	54%	5.04	40%	4.41	45%
4	♂	18	Pulmonary contusions. Minor laceration of the right kidney.	-	None	No	1.1	4.54	24%	4.45	25%	4.36	25%	4.64	24%	4.06	27%
5	♂	54	Ethyl alcohol poisoning (4.32 mg/l). Rib fractures.	Steatosis and severe non-cirrhotic liver fibrosis. Moderate diffuse myocardial fibrosis	None	Yes	2.7	5.75	47%	5.60	48%	5.57	48%	6.24	43%	5.46	49%
6	♀	24	Superficial pulmonary laceration. Rib fractures.	-	None	Yes	1.5	4.43	34%	4.85	31%	4.25	35%	6.26	24%	5.47	27%
7	♂	39	Diffuse mesentery bleeding. Superficial spleen injury. Perirenal hemorrhage. Rib fractures. Fracture of the left femur. Superficial laceration of the left lung. 100 ml of blood in both pleural cavities.	Steatosis	Kidney pallor	Unknown	0.7	6.02	12%	5.96	12%	6.05	12%	8.24	8%	7.21	10%
8	♂	51	Fracture through the 5th thoracic vertebra. Rib fractures.	-	Kidney pallor	Yes	0.9	4.92	18%	4.86	19%	4.84	19%	5.41	17%	4.73	19%
9	♀	44	Rib fractures.	-	Kidney (and possibly lung) pallor	Yes	1.7	4.97	34%	5.36	32%	4.71	36%	7.12	24%	6.23	27%
10	♂	24	Small portal vein rupture. Rib fractures. Minor pulmonary lacerations.	.-	Lung and kidney pallor	Yes	1.7	6.76	25%	6.63	26%	6.71	25%	9.52	18%	8.33	20%
11	♂	73	Minor tears of the aortic intima. Pulmonary contusions, and lacerations with 250 ml of blood-tinged fluid in the right pleural cavity. Fractures of the left humerus. Rib fractures.	Mild cardiac hypertrophy. Pulmonary emphysema.	None	No	1	4.69	21%	4.68	21%	4.70	21%	5.44	18%	4.76	21%
12	♂	64	-	Metastatic colon cancer. Pulmonary emphysema.	None	Yes	1.5	5.23	29%	5.18	29%	5.19	29%	6	25%	5.25	29%
13	♂	46	Rib fractures.	Steatosis.	Lung and kidney pallor	No	1	5.25	19%	5.22	19%	5.25	19%	6.24	16%	5.46	18%
14	♂	63	Food aspiration. Rib fractures. Clavicle fracture	Minor coronary artery disease.	Lung and kidney pallor	Yes	2	4.50	44%	4.51	44%	4.54	44%	5.36	37%	4.69	43%
15	♂	56	Rib fractures. Minor pulmonary contusions and lacerations. Ethyl alcohol poisoning (3.5 mg/l). Minor diaphragm laceration	Steatosis. Moderate coronary artery disease	Kidney pallor	No	1.8	4.96	36%	4.89	37%	4.87	37%	5.42	33%	4.75	38%
16	♀	47	Fracture of the left radius.	Steatosis	None	No	1	4.81	21%	5.21	19%	4.57	22%	6.8	15%	5.95	17%
17	♂	47	Right kidney injury. Blood-tinged fluid in the pleural cavities (250 ml right, 200 ml left). Pulmonary contusion. Rib fractures.		Lung pallor	Yes	1.2	4.74	25%	4.53	26%	4.35	28%	4.4	27%	3.85	31%
18	♂	37	Minor endocardial tear	Steatosis	None	No	1.5	6.56	23%	6.45	23%	6.55	23%	8.56	18%	7.49	20%
19	♂	20	Diffuse unmeasurable subdural and subarachnoid hemorrhage. Small spleen laceration and renal lacerations. Fracture of the left femur.	-	Subendocardial hemorrhage	No	0.5	4.34	12%	4.32	12%	4.30	12%	4.80	10%	4.20	12%

†*Circumstances indicate the decedent survived one week at home with the injuries before succumbing to them*

The measured exsanguinated blood volume was on average 1400 ml (range 500–2700 ml) and average calculated TBV ranged from 4.97 l (Firesen) to 6.34 l (“8% rule”). The lowest RBL of the observed data varied from 8% (“8% rule”) to 12% (Lemmens-Bernstein-Brodsky, Nadler-Hidalgo-Bloch and Friesen) depending on formula, while the highest varied from 43% (“8% rule”) to 54% (Friesen, Table 2). Notably, the case with the highest RBL was a decedent treated with anticoagulants (Table 2); if this case is omitted the highest RBL was 49% (“7% rule”).

**Fig. 2:**
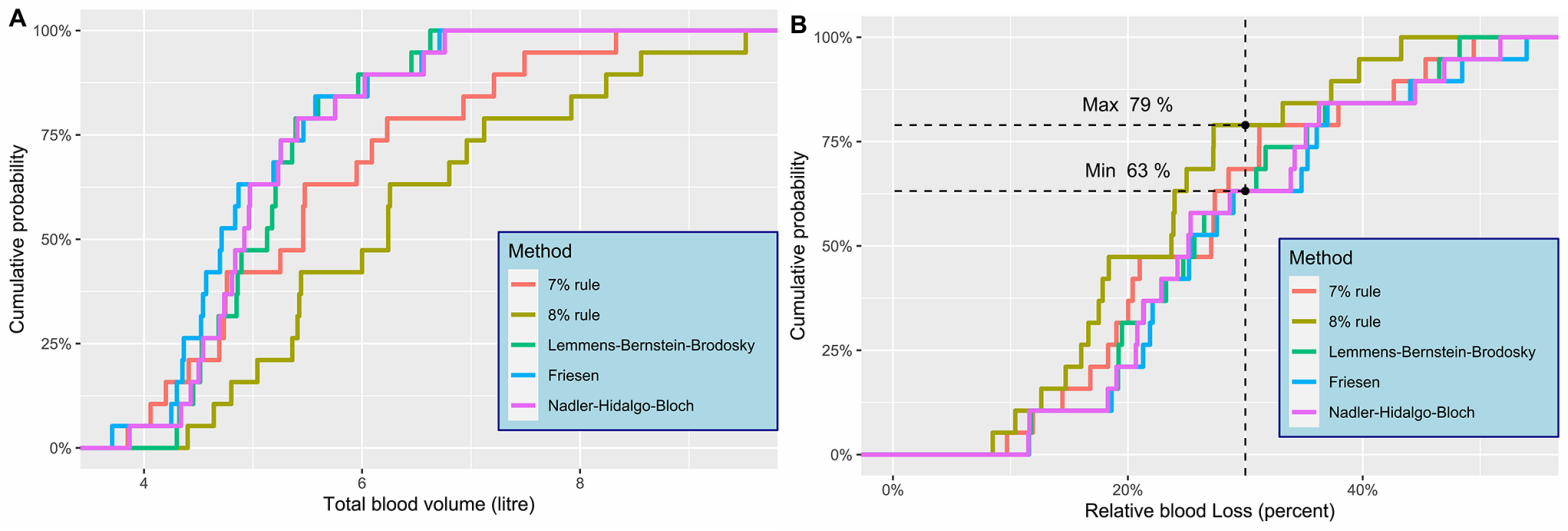
(**A**) The cumulative probability of cases reaching different total blood volumes with the different formulas used. (**B**) The cumulative probability of cases reaching different relative blood loss proportions with the different formulas used. Dashed line at 30% as well as markings of the lowest and highest cumulative probability of reaching at least 30%

**Fig. 3:**
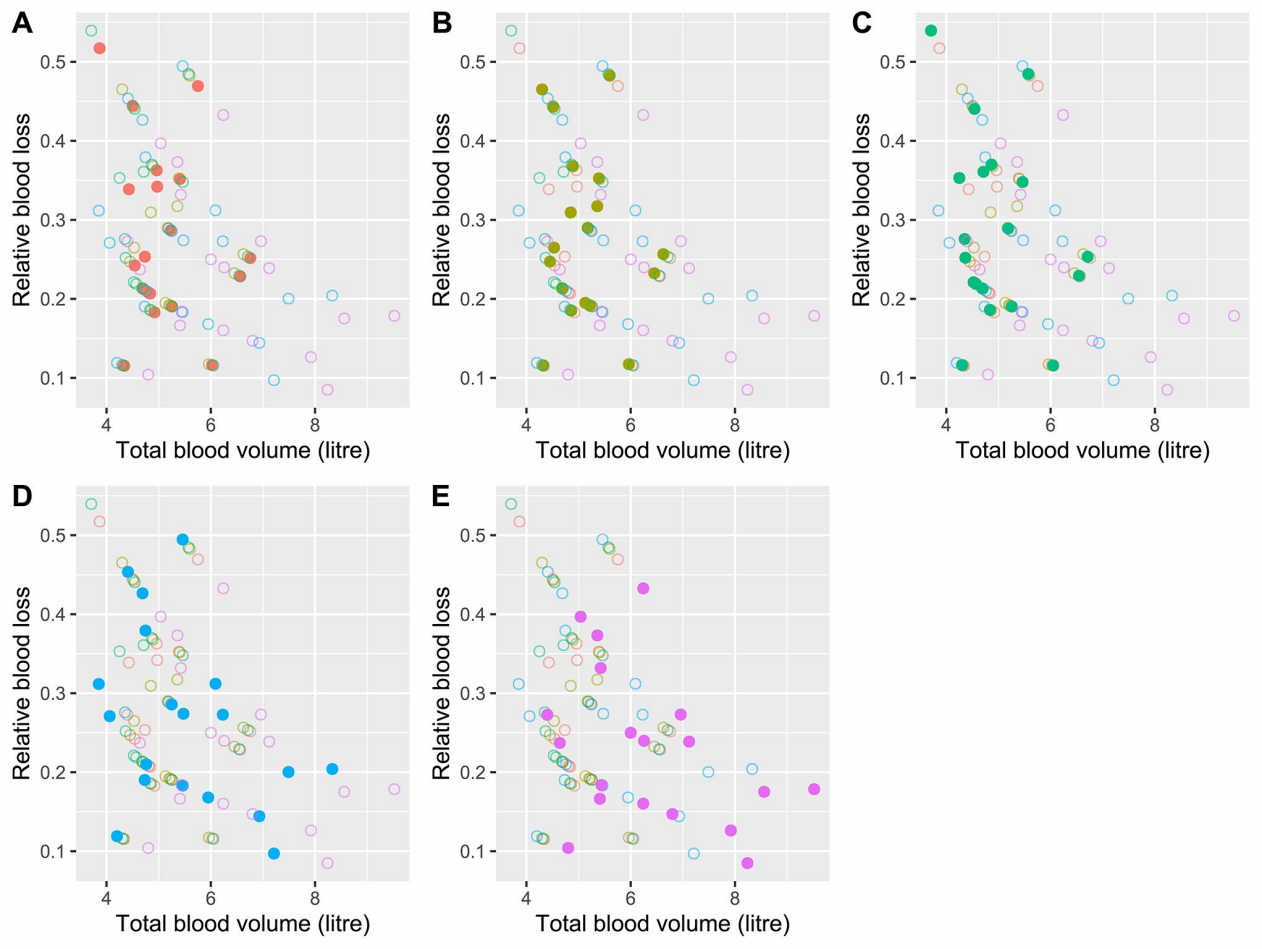
A scatter plot of relative blood loss against total blood volume using all formulas included in the study. (**A**) Nadler-Hidalgo-Bloch highlighted, (**B**) Lemmens-Bernstein-Brodsky highlighted, (**C**) Friesen highlighted, (**D**) “7% rule” highlighted, (**E**) “8% rule” highlighted

Using any of the body constitution dependent formulas 63% (12/19) cases had a RBL below 30% (Fig. 2) while for the “percentage rules” the equivalents were 68% and 79%, for the 7 and 8% rules respectively. The cases with the highest RBL were among those with the smallest calculated TBV (Fig. 3), an effect most pronounced when using the simple percentage-based formulas.

10/19 (53%) cases had undergone resuscitation attempts, 8/19 (42%) had not and in one case (5%) it was unclear. RBL among non-resuscitated cases was slightly lower (mean 22%, SD 6%) than among resuscitated cases (mean 34%, SD 11%),

Cases with known or incidental disease findings had similar RBL (Nadler-Bernstein-Bloch mean 30%, SD 13%; mean 27% SD 11% when excluding the case with anticoagulant therapy) as “healthy” cases (Nadler-Bernstein-Bloch mean 26%, SD 8%).

No cases were described as having pale lividity and eight out of nineteen cases (42%) showed no signs of hypovolemic shock at all (Table 2). The RBL was similar among cases with (Nadler-Bernstein-Bloch mean 27%, SD 13%) and cases without (Nadler-Bernstein-Bloch mean 29%, SD 9%) signs of hypovolemia.

**Fig. 4:**
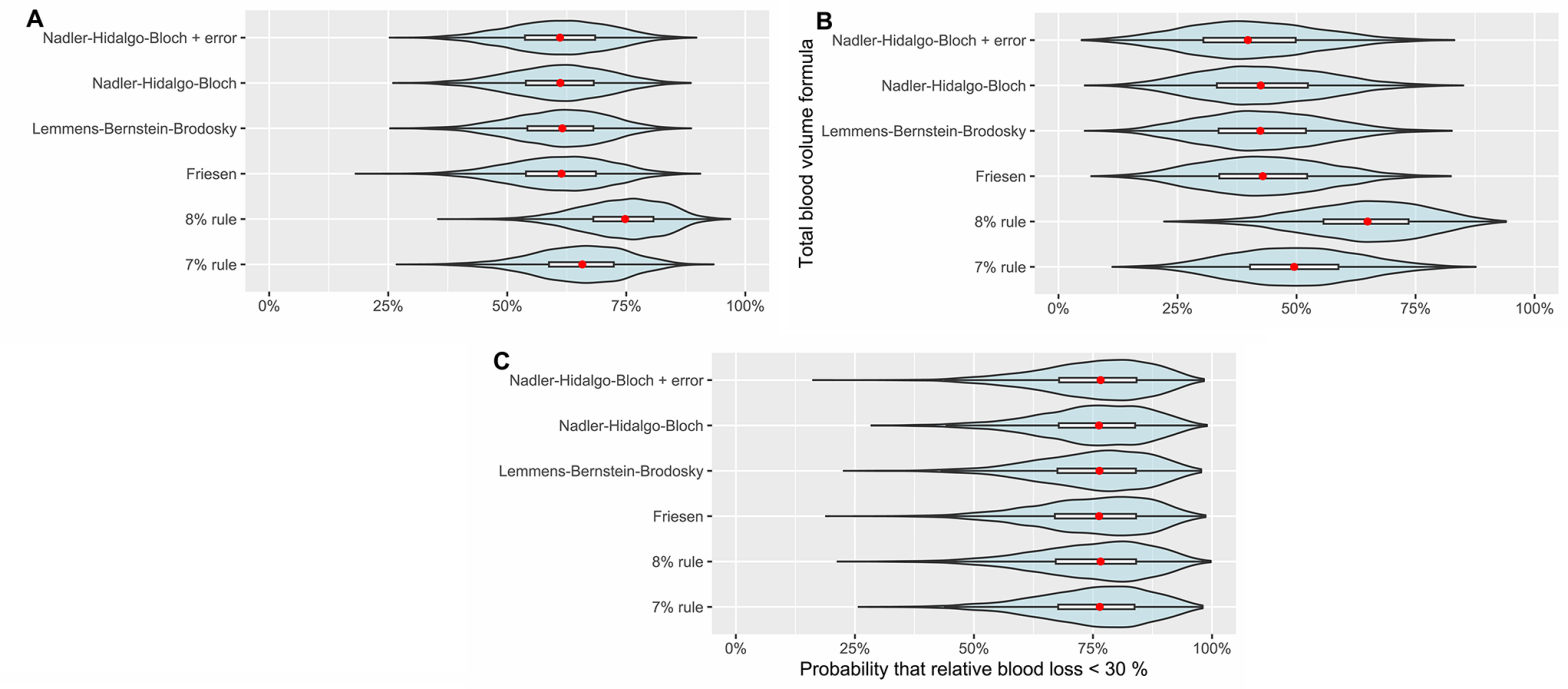
A box and violin plot of the posterior distribution of the probability of cases having a relative blood loss of less than 30% using all the included formulas for (**A**) all cases, (**B**) cases that undergone resuscitation attempts and (**C**) cases that had not undergone resuscitation attempts

**Fig. 5:**
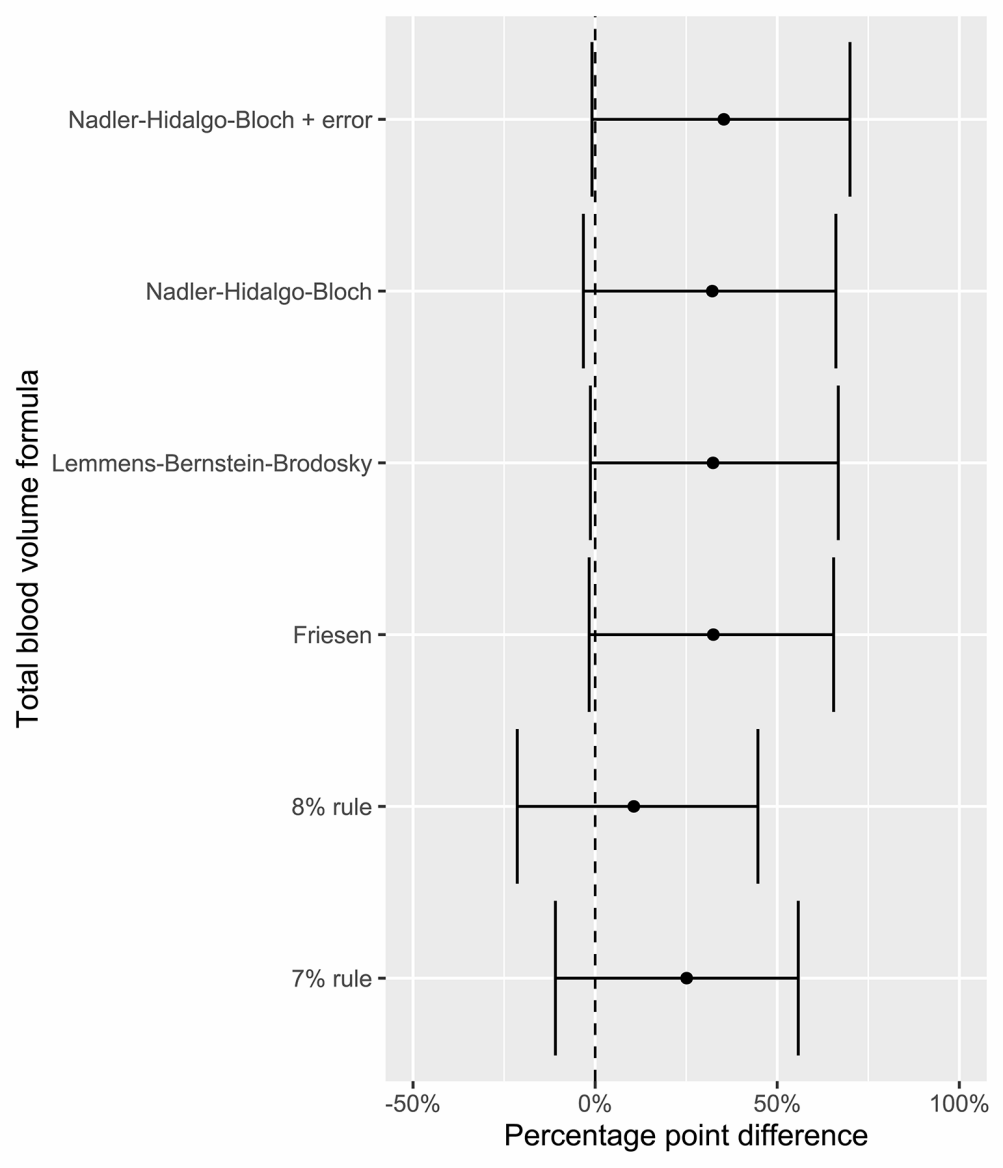
The posterior distribution of differences in probability of a relative blood loss < 30% for all included formulas between cases that had undergone a resuscitation attempt and those who had not

The posterior distribution of the probability of a case having RBL < 30% was 61% (41–80%, 95% HDPI) when using the Nadler-Bernstein-Bloch formula, with similar results among all models except the “8% rule” (Fig. 4). There was a higher mean probability of RBL < 30% among the cases who had not undergone resuscitation attempts than among those who had (Fig. 4). Using all formulas, with the exception of the “8% rule”, there was a high probability that cases that had undergone resuscitation attempts had a lower chance of RBL < 30% than cases who had not undergone resuscitation attempts (Fig. 5).

## Discussion

Our findings show that the decedents who died from isolated liver lacerations, i.e., deaths likely caused by internal bleeding only, had greatly varying levels of RBL, with a substantial proportion of cases having an RBL < 30%. Relative blood loss ranged from 8 to 54%, depending on formula used, and at 63% of the decedents did not reach a RBL classified as class III-IV haemorrhage (i.e., life-threatening) according to Advanced Trauma Life Support (ATLS^®^) [7], seemingly confirming the results of the index study where a relative blood loss of 30% was (on average) not reached in 53% of the studied fatalities [6].

Our results, taken together with the earlier study, indicate that the volume classification in ATLS^®^ considered as life-threatening may be overly conservative in assessing the severity of blood loss for medico-legal purposes. A more suitable approach would be a score-based method, which has been shown to accurately assess lethality in cases of penetrating injury cases generally [20]. However, more research is needed to explore the potential benefits and limitations in medico-legal practice.

No matter the method used, correctly identifying whether injuries are life-threatening or not is of immense importance to the legal system, as indeed there is a strong correlation between medico-legal assessments of lethality and crime classification [21]. However, many medico-legal practices (such as using the ATLS^®^ classifications) are not evidence-based, instead relying primarily on customary practice and eminence-based values [3]. We can with some emphasis now say that this particular practice should be abandoned.

We will nonetheless note here that the reader should not take this as a blanket statement that “experience” is irrelevant and that medico-legal physicians should be strictly bound to only report empirical facts. Indeed, an assessment of a particular case cannot be based on data alone, as any such statement purely based on empirical science is conditional on the assumption that the underlying model or data are relevant to the case at hand and, as such, nevertheless reflect a “subjective” probability or state of information [22]. However, the validity of any assessment must surely be improved by being informed by available scientific evidence.

Though scarcely more than noise, we note a potential downward trend wherein cases with a higher TBV had a proportionally lower IABV and as such a smaller RBL (Fig. 3). If more than simply noise, this could imply one of two things; either the autopsying pathologist looked at blood volume alone and did not actually assess the *relative* blood loss or the TBV of heavier individuals is systematically overestimated at even greater rate than that for which our measurement error model could account. However, as we have stressed, data points are few and the amount of blood in the abdominal cavity varied, and as such, this is merely speculation.

We chose isolated liver lacerations as they would likely represent “pure” haemorrhage deaths without other potential mechanisms for lethality, under the assumption that other mechanisms could work to limit the potential volume of blood loss, and thus this group would give the ATLS^®^ criteria the best chance possible to work. Surprisingly our cases do not seem to have on average higher haemorrhage volumes than those in the index study [6]. It is unclear why this would be, but the evidence does not seem to support the notion that the haemorrhages were fast nonetheless: Very few cases showed any clear signs of hypovolemic shock and none had recorded pale lividity. This unexpected finding does is in our opinion support the idea that these deaths *are* likely slower than the rapid exsanguination in, e.g., aortic rupture, and thus there was time for the homeostatic compensation to counteract hypovolemia thereby obscuring signs such as “shock kidneys”. It is of course possible that signs of hypovolemia *were* present but the physician undertaking the autopsy simply did not take note of them, though we believe that a systematic bias of this kind is less likely.

The fact that it was highly likely that there *was* a difference in how probable a RBL below 30% was when comparing cases that had undergone a resuscitation attempt to those who had not lend support to the hypothesis that resuscitation attempts indeed does influence haemorrhage volume, implying that the ATLS^®^ classifications may be especially ill suited in such cases.

The scientific value of a small case series study such as this is limited. The results are based on a small number of cases and care must be interpreted cautiously. The primary study group, isolated liver lacerations represent very rare cases, as most lethal abdominal trauma causes have multiple injuries. Further, we note the issue that the described details varied between individual forensic pathologists, making it difficult to accurately assess signs of pallor or whether the differences were merely artistic. Nevertheless, the study can be said to indeed show that there *is* a large contingent of cases that have a RBL below 30%, even when controlling for non-haemorrhagic mechanisms of death.

## Conclusions

As the relative blood loss in most deaths caused by internal bleeding in our study did not reach 30%, the results lend further support to the claim that the volume classification of hypovolemic shock described in ATLS^®^ is inappropriate for assessing the lethality of injuries in medical-legal cases.

## Declarations

Ethics

The study used only data from deceased individuals which for the purpose of the General Data Protection Regulation article 4 do not count as “personal data” and as such the study did not require ethical approval pursuant to the § 3 of the Swedish Ethical Review Act (Etikprövningslagen 2003:460).
